# AI models for the identification of prognostic and predictive biomarkers in lung cancer: a systematic review and meta-analysis

**DOI:** 10.3389/fonc.2025.1424647

**Published:** 2025-02-26

**Authors:** Hind M. AlOsaimi, Aseel M. Alshilash, Layan K. Al-Saif, Jannat M. Bosbait, Roaa S. Albeladi, Dalal R. Almutairi, Alwaleed A. Alhazzaa, Tariq A. Alluqmani, Saud M. Al Qahtani, Sara A. Almohammadi, Razan A. Alamri, Abdullah A. Alkurdi, Waleed K. Aljohani, Raghad H. Alraddadi, Mohammed K. Alshammari

**Affiliations:** ^1^ Department of Pharmacy Services Administration, King Fahad Medical City, Riyadh Second Health Cluster, Riyadh, Saudi Arabia; ^2^ Department of Medicine, Royal College of Surgeons Ireland (RCSI) University of Medicine and Health Sciences, Dublin, Ireland; ^3^ Department of Medicine, Majmaah University, Almajmaah, Saudi Arabia; ^4^ Department of Medicine, King Faisal University, Alahsa, Saudi Arabia; ^5^ Department of Medicine, King Abdulaziz University, Rabigh, Saudi Arabia; ^6^ Department of Medicine, King Faisal University, Al-Ahsa, Saudi Arabia; ^7^ Department of Medicine, King Saud Bin Abdulaziz University for Health Sciences, Riyadh, Saudi Arabia; ^8^ Department of Medicine, Taibah University, Al-Madinah Al-Munawwarah, Saudi Arabia; ^9^ Department of Medicine, King Abdulaziz University, Jeddah, Saudi Arabia; ^10^ Unaizah College of Pharmacy, Qassim University, Qassim, Saudi Arabia; ^11^ Department of Medicine, Taif University, Taif, Saudi Arabia; ^12^ Department of Medicine, Al Rayan National College of Medicine, Al-Madinah Al-Munawwarah, Saudi Arabia; ^13^ Department of Clinical Pharmacy, King Fahad Medical City, Riyadh, Saudi Arabia

**Keywords:** AI models, identification, prognostic and predictive biomarkers, lung cancer, systematic review, meta-analysis

## Abstract

**Introduction:**

This systematic review and meta-analysis aim to evaluate the efficacy of artificial intelligence (AI) models in identifying prognostic and predictive biomarkers in lung cancer. With the increasing complexity of lung cancer subtypes and the need for personalized treatment strategies, AI-driven approaches offer a promising avenue for biomarker discovery and clinical decision-making.

**Methods:**

A comprehensive literature search was conducted in multiple electronic databases to identify relevant studies published up to date. Studies investigating AI models for the identification of prognostic and predictive biomarkers in lung cancer were included. Data extraction, quality assessment, and meta-analysis were performed according to PRISMA guidelines.

**Results:**

A total of 34 studies met the inclusion criteria, encompassing diverse AI methodologies and biomarker targets. AI models, particularly deep learning and machine learning algorithms demonstrated high accuracy in predicting biomarker status. Most of the studies developed models for the prediction of EGFR, followed by PD-L1 and ALK biomarkers in lung cancer. Internal and external validation techniques confirmed the robustness and generalizability of AI-driven predictions across heterogeneous patient cohorts. According to our results, the pooled sensitivity and pooled specificity of AI models for the prediction of biomarkers of lung cancer were 0.77 (95% CI: 0.72 – 0.82) and 0.79 (95% CI: 0.78 – 0.84).

**Conclusion:**

The findings of this systematic review and meta-analysis highlight the significant potential of AI models in facilitating non-invasive assessment of prognostic and predictive biomarkers in lung cancer. By enhancing diagnostic accuracy and guiding treatment selection, AI-driven approaches have the potential to revolutionize personalized oncology and improve patient outcomes in lung cancer management. Further research is warranted to validate and optimize the clinical utility of AI-driven biomarkers in large-scale prospective studies.

## 1 Introduction

Lung cancer remains one of the most prevalent and lethal malignancies globally, posing significant challenges to public health despite advancements in diagnosis and treatment modalities (1, 2). Despite advances in therapeutic interventions such as targeted therapies and immunotherapy, the overall prognosis for lung cancer remains dismal, emphasizing the critical need for personalized treatment strategies (3). The intricate heterogeneity of lung cancer underscores the necessity for precise prognostic and predictive biomarkers to guide therapeutic strategies and improve patient outcomes (4). Traditional biomarker discovery approaches have been limited by their reliance on small sample sizes, low reproducibility, and insufficient consideration of the complex interactions within the tumor microenvironment (5). In recent years, the integration of artificial intelligence (AI) models has emerged as a promising approach for the identification and validation of biomarkers in various cancers, including lung cancer (6). AI-based methodologies offer a data-driven paradigm capable of analyzing large-scale genomic, transcriptomic, and clinical datasets to uncover novel biomarkers and elucidate underlying biological mechanisms (7, 8). Studies have demonstrated the efficacy of AI algorithms, including machine learning and deep learning techniques, in identifying prognostic biomarkers associated with survival outcomes and predicting treatment response in lung cancer patients (9–11). These methodologies leverage diverse data sources, including gene expression profiles, imaging features, and clinical variables, to generate predictive models with enhanced accuracy and generalizability (12). Despite these advancements, the translation of AI-derived biomarkers into clinical practice necessitates rigorous validation across heterogeneous patient cohorts and consideration of potential confounding factors.

The identification of robust prognostic and predictive biomarkers holds profound implications for optimizing therapeutic decision-making and advancing precision oncology in lung cancer (13). By stratifying patients based on their molecular profiles and risk profiles, clinicians can tailor treatment regimens to individualize care, thereby maximizing efficacy and minimizing toxicity (14). Moreover, prognostic biomarkers offer valuable insights into disease progression and patient prognosis, enabling timely interventions and facilitating patient counseling (15). While numerous studies have explored the potential of AI models for biomarker discovery in lung cancer, several key gaps persist in the existing literature. These include the limited reproducibility of findings across independent cohorts, the lack of consensus regarding optimal feature selection and model validation strategies, and the need for comprehensive meta-analyses to synthesize existing evidence and identify overarching trends. Additionally, the majority of studies have focused on single omics modalities or clinical variables, overlooking the potential synergies arising from integrating multi-omics data and incorporating spatial and temporal heterogeneity. In light of these considerations, the objective of this systematic review and meta-analysis was to comprehensively assess the landscape of AI-driven methodologies for discerning prognostic and predictive biomarkers in lung cancer, thereby elucidating their clinical utility and potential implications. The primary aim of the current review was to examine the performance of AI-driven models mainly focusing on key metrics such as specificity, sensitivity, and accuracy. A meta-analysis was performed of identified studies to assess the overall performance and reproducibility of AI-derived biomarkers and to identify existing challenges and opportunities for future research in this rapidly evolving field. By examining these key performance metrics, we assessed the reliability of these AI models and their potential to serve as non-invasive alternatives to conventional diagnostic methods in healthcare system and outline recommendations and prospects.

## 2 Materials and methods

### 2.1 Study design

This systematic review and meta-analysis were conducted in accordance with the Preferred Reporting Items for Systematic Reviews and Meta-Analyses (PRISMA) guidelines to ensure comprehensive and transparent reporting of the study methodology and findings.

### 2.2 Search strategy

A comprehensive literature search was performed in electronic databases including PubMed/MEDLINE, Embase, Web of Science, Google Scholar, Science direct and Scopus from inception to date. The search strategy utilized a combination of medical subject headings (MeSH) terms and keywords related to “lung cancer,” “biomarkers,” “artificial intelligence,”, “deep learning” and “machine learning.” The search strategy was adapted to the syntax and specifications of each database to maximize sensitivity while maintaining relevance.

### 2.3 Inclusion and exclusion criteria

Studies were included in the review if they met the following criteria:

1. Investigated the use of AI models for the identification of prognostic or predictive biomarkers in lung cancer.
2. Included human participants diagnosed with lung cancer.
3. Published in English language.
4. Original research articles reporting primary data.
5. Studies with full-text availability.

Studies were excluded if they were:

1. Review articles, editorials, conference abstracts, or letters.
2. Studies focusing solely on non-human subjects.
3. Studies not relevant to the objectives of this review.

### 2.4 Study selection process

Two independent reviewers screened the titles and abstracts of retrieved articles to identify potentially eligible studies. Full-text articles were then assessed for eligibility based on the inclusion and exclusion criteria. Any disagreements between reviewers were resolved through discussion or consultation with a third reviewer.

### 2.5 Data extraction

Data extraction was performed using a standardized data extraction form, including the following information:

1. Study characteristics: authors, publication year, study design, sample size.
2. Patient demographics: age, gender, histological subtype, cancer stage.
3. AI model details: type of AI algorithm, input data types (e.g., genomic, imaging), model performance metrics (sensitivity, specificity, accuracy).
4. Biomarkers identified: prognostic or predictive biomarkers, associated outcomes.
5. Validation methods: internal or external validation, cross-validation techniques.

Outcomes of study: main findings.

### 2.6 Quality assessment

**Figure 1:**
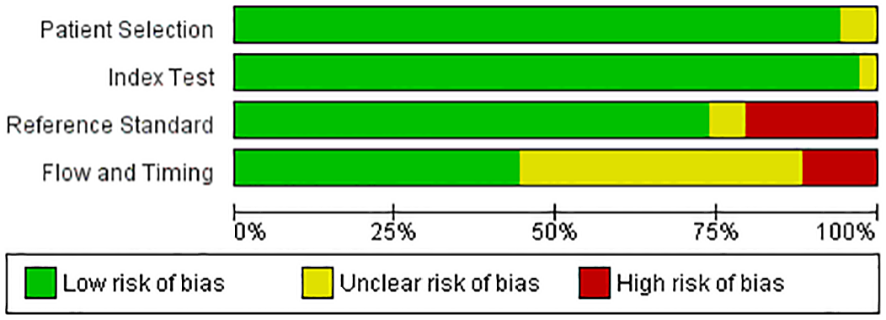
Quality assessment of included studies using QUADAS-2 tool (42).

The methodological quality and risk of bias of included studies were assessed independently by two reviewers using validated tools appropriate for the study design. Risk of bias was assessed using relevant tools i.e., QUADAS-2 tool. Any discrepancies were resolved through discussion or consultation with a third reviewer. Studies were evaluated based on criteria such as sample representativeness, outcome ascertainment, statistical analysis methods, and reporting transparency (**Figure 1**).

### 2.7 Data synthesis and meta-analysis

A narrative synthesis of included studies was conducted to summarize key findings, including AI model performance, biomarkers identified, and clinical implications. The statistical analyses were carried out using R software (Version 4.3.0, Vienna, Austria). Libraries such as meta and metaphor were used to calculate the key metrics of AI models such as sensitivity, specificity, and accuracy. The random effects model was employed to evaluate the pooled sensitivity and specificity of the AI models that were involved in the prediction and prognosis biomarkers of lung cancer. Additionally, the heterogeneity was assessed among the included articles using the chi-square test and I2 index statistics.

### 2.8 Ethical considerations

As this study involved the analysis of existing literature, ethical approval was not required. However, ethical principles such as confidentiality and respect for intellectual property rights were upheld throughout the review process.

## 3 Results

**Figure 2:**
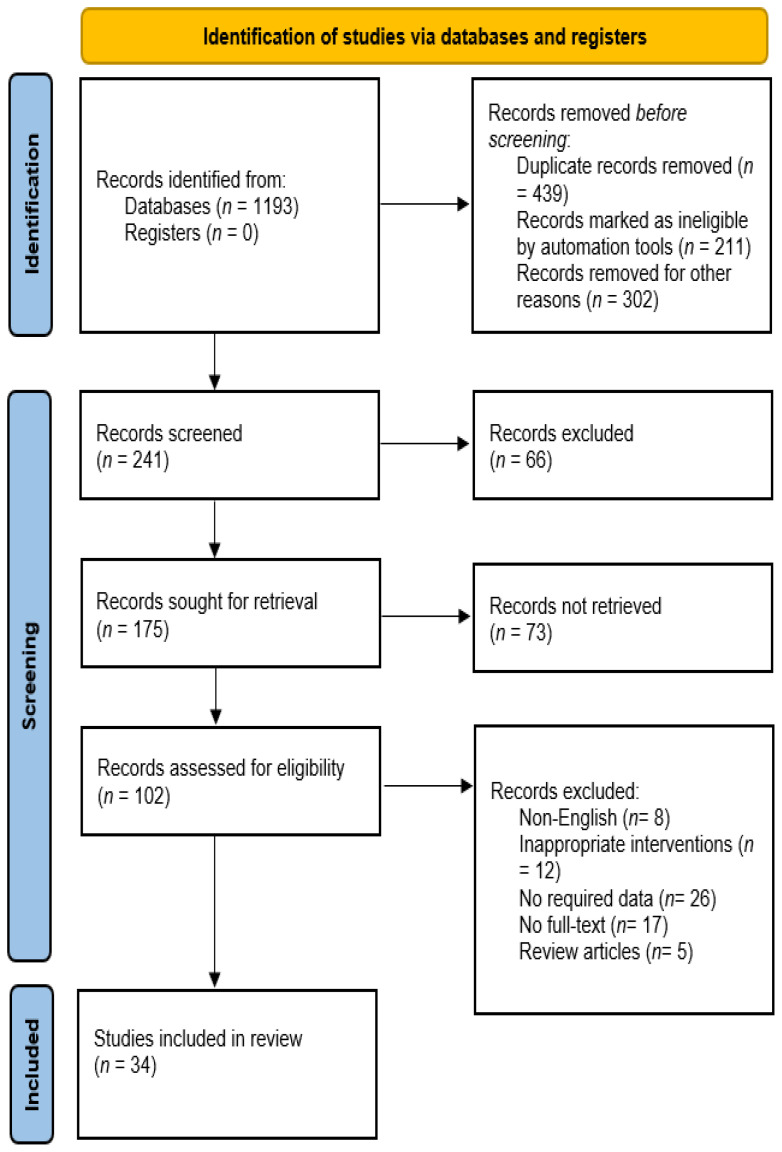
Flow chart of included studies.

The PRISMA chart in **Figure 2** depicts the process of selecting studies for a systematic review. In this review, the search identified 1,193 records from databases and zero records from registers. After removing duplicates and records ineligible based on automation tools or other reasons, 241 records remained. Of the 241 records, reviewers excluded 66 studies for various reasons. After this process, 175 studies were sought for retrieval, but 73 were not retrieved. This left 102 studies to be assessed for eligibility and studies excluded not being in English, investigating inappropriate interventions, lacking required data, or being review articles. Ultimately, 34 studies were included in the review. Overall, the PRISMA chart demonstrates a rigorous process for selecting studies that met the inclusion criteria for the systematic review.

The **Table 1** provides a comprehensive overview of studies employing AI models for the identification of predictive and prognostic biomarkers in lung cancer. The included studies predominantly utilized retrospective designs, with sample sizes ranging from small cohorts of fewer than 100 patients to larger cohorts exceeding 3,000 individuals. The studies primarily focused on non-small cell lung cancer (NSCLC), encompassing various histological subtypes such as adenocarcinoma and squamous cell carcinoma. The biomarkers investigated included Programmed death-ligand 1 (PD-L1), Epidermal growth factor receptor (EGFR), Anaplastic lymphoma kinase (ALK), Kirsten rat sarcoma (KRAS), and others associated with tumor proliferation and therapeutic response. DL models, particularly CNNs, were frequently utilized due to their ability to process complex data such as imaging and genomic profiles. The performance of AI models was evaluated using sensitivity, specificity, and accuracy measures. Overall, the models exhibited high accuracy in predicting biomarker status, with some variability observed across different studies and validation methods. Internal and external validation techniques, including cross-validation and independent testing sets, were employed to assess the generalizability and robustness of AI models. Internal validation within the same dataset was most commonly utilized, followed by external validation using independent datasets. External validation was found to provide stronger evidence of model generalizability, particularly in studies utilizing multicenter or population-based cohorts. Future research should prioritize external validation to confirm that AI models maintain predictive accuracy across diverse patient populations and data sources, thereby increasing their reliability for clinical implementation. The findings underscore the potential of AI-driven approaches in enhancing diagnostic and therapeutic decision-making in lung cancer management. AI models demonstrated significant potential in facilitating non-invasive assessment of biomarker expression, aiding in patient stratification, treatment selection, and prognostic evaluation. By accurately predicting biomarker status using non-invasive methods such as imaging or blood tests, AI models can help clinicians tailor treatment strategies to individual patients, maximizing therapeutic efficacy while minimizing unnecessary interventions and adverse effects. Furthermore, the ability of AI models to analyze large-scale genomic and clinical data sets provides insights into the underlying molecular mechanisms driving tumor progression and treatment response, paving the way for more targeted and personalized approaches to lung cancer management. As such, the integration of AI technologies into clinical practice has the potential to revolutionize patient care by improving diagnostic accuracy, treatment outcomes, and overall survival rates in lung cancer patients.

**Table 1:** AI models for the identification of predictive and prognostic biomarkers in lung cancer.

Author and year	Study design	Sample size	Type of lung cancer	Biomarker analyzed	AI model used	Type of data	Sensitivity	Specificity	Accuracy	Validation method	Outcomes
Cheng et al., 2022 (12)	Retrospective study	1288	Lung adenocarcinoma and lung squamous cell carcinoma	PD-L1	DL	WSIs	95.2%	96.7%	96.3%	Cross-validation	AI-assisted diagnostic tools showed promising results in identifying PD-L1 expression, thus improving the efficiency of experts.
Wu et al., 2022 (7)	Retrospective study	239 slides	Non-small cell lung cancer	PD-L1	DL	WSIs	86%	96.4%	93.2%	NA	DL model provides non-invasive method for assessment of PD-L1 expression in NSCLC patients.
Tian et al., 2021 (9)	Retrospective study	939 patients	Non-small cell lung cancer	PD-L1	CNN	–	–	–	–	Internal validation	DL model helps to assess the PD-L1 expression and to improve the therapeutic outcomes in response to immunotherapy.
Wang et al., 2021 (13)	Retrospective study	1262 patients	Non-small cell lung cancer	EGFR and PD-L1	CNN	CT images	Training set: 74% Validation set: 48% Test set: 43%	Training set: 93% Validation set: 84% Test set: 82%	Training set: 90% Validation set: 78% Test set: 75%	Internal validation	A non-invasive and effective model was employed to predict EGFR mutation and PD-L1 expression status and help in screening patients before invasive techniques.
Mu et al., 2021 (10)	Retrospective study	284 patients	Non-small cell lung cancer	PD-L1	CNN	FDG-PET/CT images	Training set: 84.7% Validation set: 77.4% Test set: 68.7%	Training set: 80.4% Validation set: 81.4% Test set: 89.1%	Training set: 81.6% Validation set: 78.4% Test set: 77.6%	External validation	CNN model is considered as substitute for the assessment of PD-L1 to guide individual pretherapy decisions.
Tan et al., 2022 (16)	Retrospective study	2553 patients	Non-small cell lung cancer	EGFR	DL and ML	–	Training set: 83.5% Testing set: 85.6%	Training set: 67.7% Testing set: 68%	Training set: 57.8% Testing set: 63.8%	Internal validation	The proposed AI model achieved high sensitivity, specificity, and accuracy for the predicting EGFR in NSCLC patients.
Adetiba et al., 2011 (14)	Retrospective study	305 patients	Non-small cell lung cancer	EGFR	ANN	–	–	–	–	Internal validation	Using the ANN approach, we achieved predictions with minimal errors and provides a basis for early diagnosis of NSCLC.
Morgado et al., 2021 (11)	Retrospective study	96 patients	Non-small cell lung cancer	EGFR	ML	CT images	SVM 61.5% Elastic net 61.1% Logistic regression 69.9% Random Forest 68.8%	SVM 68.5% Elastic net 71.5% Logistic regression 74.3% Random Forest 72.1%	–	Cross-validation	Random forest model showed the promising results in identifying EGFR biomarkers in cancer patients.
Haim et al., 2022 (17)	Retrospective study	59 patients	Non-small cell lung cancer	EGFR	DL	MRI images	68.7%	97.7%	89.9%	Cross-validation	DL-based model provides the non-invasive method to detect EGFR with high accuracy and it should be validated in large prospective cohorts.
Zhang et al., 2021 (18)	Retrospective study	134 patients	Non-small cell lung cancer	EGFR, KRAS, ERBB2, and TP53	ML	CT images	EGFR 63% KRAS 93% ERBB2 100% TP53 80%	EGFR 83% KRAS 41% ERBB2 65% TP53 66%	EGFR 73% KRAS 47% ERBB2 69% TP53 72%	Cross-validation	ML-based 3D radiomics can detect the presence of EGFR, KRAS, ERBB2, and TP53 mutations in patients with NSCLC with high accuracy, sensitivity, and specificity.
Rossi et al., 2021 (19)	Retrospective study	109 patients	Non-small cell lung cancer	EGFR	ML	CT images	53.5%	96%	84.7%	External validation	ML model enables the precise identification of EGFR-mutant patients with the accuracy of 84.7%.
Wang et al., 2019 (20)	Retrospective study	844 patients	Lung adenocarcinoma	EGFR	DL	CT images	Primary 76.8% Validation 72.2%	Primary 79.0% Validation 75.4%	Primary 77.0% Validation 73.8%	Cross-validation	The proposed DL model provides a non-invasive method to predict EGFR mutation status, which can be utilized routinely CT diagnosis.
Nair et al., 2020 (21)	Retrospective study	50 patients	Non-small cell lung cancer	EGFR	ML	FDG-PET/CT images	FGD-PET 76% CT images 84%	FGD-PET 66% CT images 73%	FGD-PET 71% CT images 78%	Cross-validation	ML model can help to identify tumors with mutations in EGFR and it could be valuable for the pretreatment evaluation and prognosis in precision therapy.
Qin et al., 2020 (22)	Retrospective study	320 patients	Lung carcinoma	EGFR	ANN	CT images	Model 1 85.2% Model 2 75.0% Model 3 80.7% Model 4 90.0%	Model 1 83.9% Model 2 83.3% Model 3 78.6% Model 4 73.3%	Model 1 84.4% Model 2 80.0% Model 3 79.5% Model 4 80.0%	Internal validation	ANN models have the potential to increase the consistency, accuracy, and assist the healthcare professionals in early diagnosis of lung cancer.
Le at al., 2021 (23)	Retrospective study	161 patients	Non-small cell lung cancer	EGFR and KRAS	ML	LDCT images	EGFR 65.2% KRAS 55.6%	EGFR 88.2% KRAS 95.4	EGFR 83.6% KRAS 86%	Cross-validation	Anon-invasive ML-based model can help robustly in predicting EGFR and KRAS mutations in NSCLC patients.
Hao et al., 2022 (24)	Retrospective study	193 patients	Non-small cell lung cancer	ALK	ML	CT images	–	–	LR 86.9% Adaboost 80.8% Decision Tree 81.2% XGBoost 84.2% SVM 84.9%	Cross-validation	This study reported that ALK rearrangement status could be accurately predicted using an ML-based classification model one the basis of clinical data and CT images.
Terada et al., 2022 (25)	Retrospective study	208 patients	Non-small cell lung cancer	ALK	DL	WSIs	73%	73%	–	Internal validation	DL model can serve us as non-invasive tool to identify ALK mutation in NSCLC patients.
Ma et al., 2020 (26)	Retrospective study	140 patients	Lung adenocarcinoma	ALK	ML	CT images	71.4%	82.1%	78.5%	Cross-validation	ML-radiogenomics classifier can help in identifying adenocarcinoma ALK rearrangement status, which may be cost-effective substitute for traditional invasive ALK status test.
Mahajan et al., 2023 (27)	Retrospective study	117 patients	Non-small cell lung cancer	EGFR and ALK	CNN	MRI images	–	–	76%	Internal validation	Both DL and semantic features exhibited comparable accuracy in categorizing the EGFR mutation and ALK rearrangement
Song et al., 2020 (28)	Retrospective study	335 patients	Lung adenocarcinoma	ALK	CNN	CT images	Training set: 100% Validation set: 70%	Training set: 99% Validation set: 80%	Training set: 100% Validation set: 76%	Cross-validation	The radiomics-based ML model can potentially serve as a non-invasive tool to detect ALK mutation in patients with lung adenocarcinoma.
Chang et al., 2021 (29)	Retrospective study	562 patients	Lung adenocarcinoma	ALK	ML	PET/CT images	Training set: 57.9% Testing set: 62.5%	Training set: 94.3% Testing set: 94%	Training set: 83.7% Testing set: 86%	Cross-validation	PET/CT radiomics-based ML model provides non-invasive diagnostic method to help diagnose ALK mutation status for lung adenocarcinoma patients.
Wang et al., 2022 (30)	Prospective study	978 patients	Lung cancer	EGFR	FAIS	CT images	–	–	72.3%	Internal validation	FAIS provides a non-invasive method to detect EGFR genotype and identify patients with an EGFR mutation.
Shiri et al., 2020 (31)	Retrospective study	211 patients	Non-small cell lung cancer	EGFR and KRAS	ML	PET/CT images	KRAS: 18.5%	KRAS 98.9%	KRAS 79.8%	Cross-validation	The proposed approach achieved cutting-edge outcomes in predicting EGFR and KRAS mutation status in NSCLC patients.
Dong et al., 2021 (32)	Retrospective study	363 patients	Non-small cell lung cancer	EGFR and KRAS	MMDL	CT images	EFGR: 78.2% KRAS: 71.8%	EFGR: 81.3% KRAS: 74.2%	EFGR: 86.5% KRAS: 78.9%	Internal validation	The DL model provides the latest methods in predicting EGFR and KRAS mutations in NSLC patients.
Gu et al., 2019 (33)	Retrospective study	245 patients	Non-small cell lung cancer	KI-67	ML	PET/CT images	72.6%	66.6%	–	Cross-validation	ML-based CT radiomics classifier plays a substantial role in assessing cell proliferation and predicting Ki-67 expression.
Hong et al., 2021 (34)	Retrospective study	478 patients	Lung adenocarcinoma	STK-11	CNN	Histopathology images	–	–	85.5%	Internal validation	CNN model can help to identify STK11 mutations based on histopathology slides with high accuracy.
Li et al., 2020 (35)	Retrospective study	438 patients	Non-small cell lung cancer	EGFR	DL	CT images	SVM: 80% Random Forest: 83% Naïve Bayes 81% Logistic regression 83% Neural network: 82%	SVM: 67% Random Forest: 55% Naïve Bayes 64% Logistic regression 63% Neural network: 64%	SVM: 74% Random Forest: 71% Naïve Bayes 74% Logistic regression 75% Neural network: 75%	External validation	The radiomics shows promising result to predict EGFR mutation status in NSCLC patients.
Lu et al., 2024 (36)	Retrospective study	274 patients	Non-small cell lung cancer	EGFR T790M	ML	CT images	Random forest: 86% Combined models 76%	Random forest: 85% Combined models 75%	Random forest: 78% Combined models: 77%	Cross-validation	A convenient and non-invasive radiomics-based ML model can not only predict EGFR-mutation at the time of diagnosis, but also can aid in targeted treatment planning for NSCLC patients.
He et al., 2022 (37)	Retrospective study	758 patients	Lung adenocarcinoma	EGFR	ML	CT images	–	–	K-nearest neighbor: 81% Random forest: 83% LGBM: 88% SVM: 83%	Cross-validation	The proposed ML model effectively predict the EGFR mutation status of NSCLC patients.
Jia et al., 2019 (38)	Retrospective study	503 patients	Lung adenocarcinoma	EFGR	Random forest	CT images	60.6%	85.1%	–	Internal validation	The radiomics play an important role in predicting EFGR biomarker in lung adenocarcinoma patients.
Zhao et al., 2019 (39)	Retrospective study	579 patients	Lung adenocarcinoma	EGFR	CNN	CT images	Model 1 85% Model 2 90.3% Model 3 67.7% Model 4 71%	Model 1 52.8% Model 2 49.1% Model 3 66% Model 4 58.5%	Model 1 70.4% Model 2 71.3% Model 3 67% Model 4 65.2%	Cross-validation	The proposed DL model predicts EGFR-mutant of lung adenocarcinomas accurately that help clinical decision-making by identifying eligible patients for therapy.
Lim et al., 2022 (40)	Retrospective study	312 patients	Non-small cell lung cancer	PD-L1	ML	PET/CT images	Naïve bayes 75.3% Neural network 68.1% Random forest 66.2%	Naïve bayes 58.2% Neural network 64.3% Random forest 63.5%	–	Cross-validation	The proposed non-invasive approach can help the healthcare professionals to identify tumors with positive PD-L1 expression.
Wang et al., 2022 (41)	Retrospective study	3816 patients	Non-small cell lung cancer	EGFR and PD-L1	DL	CT images	For EGFR 83.2% For PD-L1 75.8%	For EGFR 78.3% For PD-L1 82.9%	For EGFR 80.5% For PD-L1 82.4%	Internal validation	The DL-based model exhibits promising results for identifying gene status and assessing the genotypes that will help the experts in screening of NSCLC.
Hu et al., 2023 (15)	Retrospective study	159 patients	Non-small cell lung cancer	KI-67	DL	PET/CT images	–	90.2%	82.2%	–	A non-invasive and reliable DL-based FDG-PET/CT radiomics helps in assessing the malignancy and prognosis of NSCLC.

EGFR, Epidermal growth factor receptor; PD-L1, Programmed death-ligand 1; CNN, Convolutional neural network; DL, Deep learning; ML, Machine learning; ANN, Artificial neural network; NSCLC, Non-small cell lung cancer; KI-67, Antigen Kiel-67; ALK, Anaplastic lymphoma kinase; KRAS, Kirsten rat sarcoma; SVM, Support vector machine; PET, Positron emission tomography; CT, Computed tomography; FDG, Fluorodeoxyglucose; FAIS, Fully automated artificial intelligence system; MMDL, Multi-channel and multi-task deep learning model; PET, Positron emission tomography; CT, Computed tomography; WSIs, Whole slide images; LDCT, Low-dose computed tomography; FDG, Fluorodeoxyglucose.

**Figure 3:**
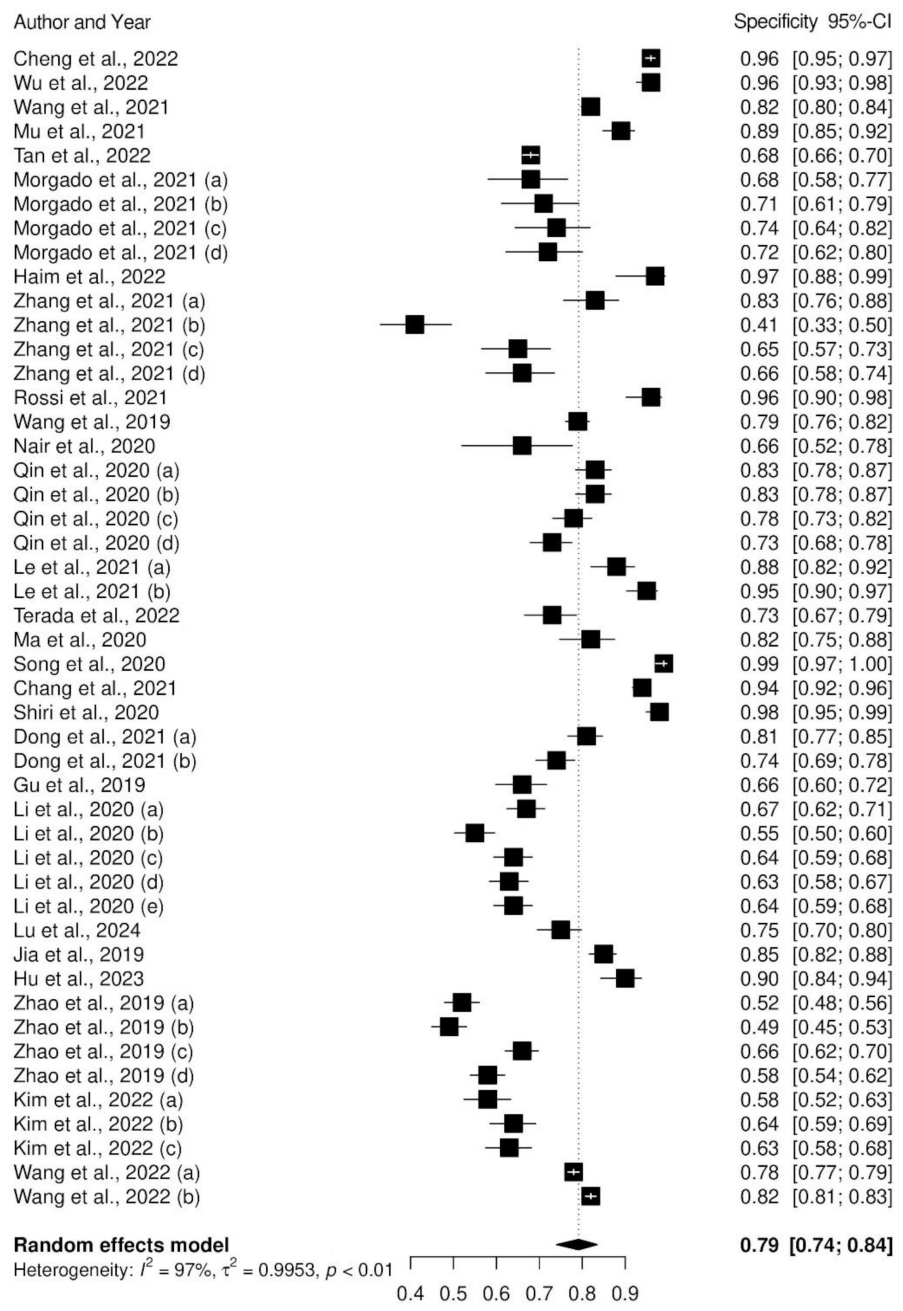
Forest plot of pooled specificity of AI models.

**Figure 4:**
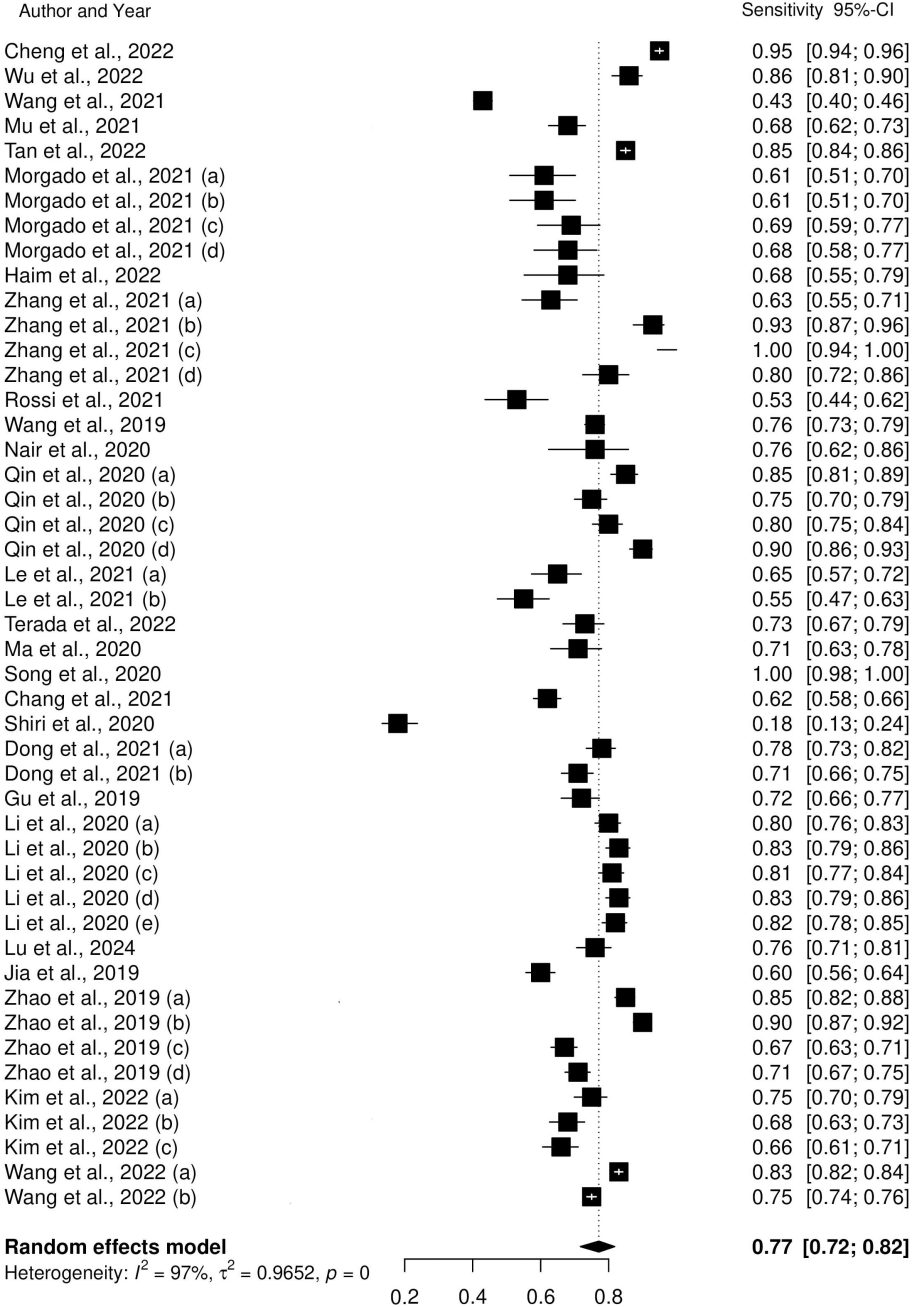
Forest plot of pooled sensitivity of AI models.

The forest plots of the pooled sensitivity and specificity of AI-assisted diagnostic system for the identification of biomarkers in lung cancer are presented in **Figures 3** and **4**. According to our results, the pooled sensitivity and pooled specificity of AI models for the prediction of biomarkers of lung cancer were 0.77 (95% CI: 0.72 – 0.82) and 0.79 (95% CI: 0.78 – 0.84). These findings suggest that AI models such as machine learning and deep learning models exhibit a high level of accuracy for the early detection of prognostic and predictive biomarkers in lung cancer. While heterogeneity was observed across included studies due to variations in study design, sample sizes, and AI model specifications, a formal subgroup analysis was not conducted. Given the diversity in AI model types (e.g., CNN, SVM, ANN), data types (e.g., imaging, genomic), and biomarker targets (e.g., EGFR, PD-L1), subgrouping studies could reduce statistical power and risk overinterpretation of the results. Instead, we qualitatively discussed these factors as sources of variability, offering insights into how they may influence model performance and generalizability. This approach allowed for a more comprehensive understanding without the confounding effects of subgrouping diverse studies.

## 4 Discussion

This systematic review scrutinizes the various methods of AI models for identifying the predictive and prognostic biomarkers in lung cancer. This review particularly focused on studies carried out within the past few years, mainly from 2010 – till date. The primary aim of the current review was to examine the performance of AI-driven models mainly focusing on key metrics such as specificity, sensitivity, and accuracy. By examining these key performance metrics, we assessed the reliability of these AI models and their potential to serve as non-invasive alternatives to conventional diagnostic methods in healthcare system and outline recommendations and prospects. This review reported that AI models for the identification of predictive and prognostic biomarkers in lung cancer demonstrated a high level of accuracy with pooled sensitivity and specificity values of 0.77 (95% CI: 0.72 – 0.82) and 0.79 (95% CI: 0.74 – 0.84), respectively. AI models played a substantial role in the field of cancer research in the existing literature. Most of the included studies have focused mainly on deep learning models (7, 16, 25). Notably, the Convolutional Neural Network (CNN) is the commonly used deep learning model for the identification of predictive and prognostic biomarkers in lung cancer (10, 27, 28). Several studies documented that CNN exhibited strong predictive performance in advanced stages of NSCLC (5, 10). Neural networks (NN) and Artificial Neural Networks (ANN) have also been used extensively in the literature (14, 22, 40). Morphologically, ANN plays a crucial role in differentiating benign from malignant tumor cells and in the identification of pulmonary nodules from computed tomography chest images (43, 44). Apart from Deep learning models, other techniques such as Support vector machine (SVM), Random Forest (RF), and Naïve Bayes are used extensively for the identification of cancer biomarkers (35, 40). Another systematic review reported similar results as the deep learning model was utilized to identify prognostic biomarkers in ovarian cancer (45, 46). Although the use of AI-based models in healthcare settings is promising, the generalizability still depends on the validity. Among 34 included studies, 13 studies performed cross-validation to assess the effectiveness and reliability of the AI model (11, 12, 17, 18, 20, 21, 23, 24, 26, 28, 29, 31, 33, 36, 37, 39, 40).

Most of the studies proposed the AI-based models for the identification of Epidermal growth factor receptor (EGFR) biomarker in non-small cell lung cancer (NSCLC), followed by Programmed death-ligand 1 (PD-L1) and anaplastic lymphoma kinase (ALK). Both machine learning and deep learning models can identify EGFR-mutant patients in various training and validation sets with great accuracy, especially after data optimization (19, 22). Haim et al., extracted the data from the limited number of NSCLC patients, and the DL approach was employed to categorize the patients in accordance with their EGFR mutation status. Lu and his colleagues designed a radiomic-based ML model that exhibited high accuracy in predicting the presence of EGFR T790M mutations using CT images at the time of diagnosis which can aid in targeted treatment planning for NSCLC patients (36). These results provided a sensitivity of 68.7%, a specificity of 97.7%, and a specificity of 89.8% for the identification of a positive EGFR mutation status (17). Moreover, PD-L1 is also considered as a crucial predictive biomarker of NSCLC response to immunotherapy (47). AI-assisted diagnostic models provide a non-invasive procedure to predict high PD-L1 expression of lung cancer and to infer the therapeutic outcomes in response to immunotherapy (9, 12, 48). Therefore, the accurate and efficient procedure for the evaluation of PD-L1 expression is a paramount for developing a reliable predictive marker of response (49). Cheng and his colleagues proposed AI models that exhibit notable key performance metrics such as sensitivity, specificity, and accuracy, particularly at the 1% cut-off value in evaluating the PD-L1 expression in tumor cells (12).

The current studies highlight several future aspects for future exploration in the field of biomarker discovery. One of the aspects involves the development of feature selection approaches that surpass the limitations of existing methods and could help in identifying predictive and prognostic biomarkers correctly (50). Moreover, to improve signature gene identification associated with biomarkers, non-linear methods should be developed that incorporate deep learning algorithms, such as DeepSurv (51). Another aspect involves the recommendation tackling the treatment effects on the basis of biomarker identification that not only improves the current identification methods but also emphasizes on the identification of predictive biomarkers (46). Lastly, incorporating additional independent or external cohorts plays a substantial role in conducting comprehensive evaluation into the progression, diagnosis, and treatment of lung cancer.

The current review has set limitations. Though AI models have marked their significance in the field of lung cancer prediction research, the researchers faced numerous challenges that need to be addressed. One of the common challenges for most of the included studies was inadequate data to train the model. A small sample size was included in the training as well as test dataset which did not authenticate the efficacy of the proposed AI model. Likewise, retrospective data can introduce biases that may not reflect real-world clinical settings, thereby limiting the generalizability of AI models. Additionally, we excluded the studies that were not mainly focused on biomarkers of lung cancer to maintain the quality and reliability of this systematic review. Most of these non-cancer biomarkers were associated with other applications and disorders such as metabolic and cardiovascular disorders, and the detection of these biomarkers demands further investigation. The data from recent studies were extracted so that current technologies should be discussed, and challenges should be addressed. Moreover, our search items were limited to the identification of predictive and prognostic biomarkers in lung cancer. We acknowledge the inclusion criteria in this review may affect the conclusions drawn from the studies included. However, the exclusion criteria were considered carefully by two independent experts. Therefore, this review aimed to focus on the identification of predictive and prognostic biomarkers in lung cancer.

The key takeaways from our review underscore the promising role of AI models in advancing non-invasive assessment of lung cancer biomarkers, with potential to reduce dependency on traditional biopsy methods in certain contexts. While AI models show high sensitivity and specificity in predicting biomarkers like EGFR and PD-L1, their real-world application requires rigorous validation across diverse populations. Our analysis also points to the need for prospective studies and the integration of multi-omics data to enhance model accuracy and clinical relevance. Standardized protocols in AI model development, including uniform definitions for data input and validation metrics, to facilitate comparability across studies. Ultimately, AI models could serve as valuable adjuncts in personalized lung cancer care, improving early detection, treatment planning, and patient outcomes.

## 5 Conclusions

This review focused on the application of AI models for identifying the predictive and prognostic biomarkers in lung cancer, mainly emphasizing the use of deep learning (DL) and machine learning (ML) models. Most of the studies developed models for the prediction of EGFR, followed by PD-L1 and ALK biomarkers in lung cancer. The pooled sensitivity and specificity values of 0.77 (95% CI: 0.72 – 0.82) and 0.79 (95% CI: 0.74 – 0.84) showed the potential of AI models for identifying true positive and true negative cases. Despite the observed heterogeneity found, our results highlight the need for the application of AI models in the prediction of biomarkers in lung cancer. Therefore, there is a need for continued research and validation in this field so that healthcare professionals will benefit from the integration of AI models in clinical practice.

## Data Availability

The original contributions presented in the study are included in the article/supplementary material. Further inquiries can be directed to the corresponding authors.
